# First Line Androgen Deprivation Therapy vs. Chemotherapy for Patients With Androgen Receptor Positive Recurrent or Metastatic Salivary Gland Carcinoma—A Retrospective Study

**DOI:** 10.3389/fonc.2019.00701

**Published:** 2019-08-02

**Authors:** Paul V. Viscuse, Katharine A. Price, Joaquin J. Garcia, David J. Schembri-Wismayer, Ashish V. Chintakuntlawar

**Affiliations:** ^1^Department of Internal Medicine, Mayo Clinic, Rochester, MN, United States; ^2^Division of Medical Oncology, Mayo Clinic, Rochester, MN, United States; ^3^Department of Laboratory Medicine and Pathology, Mayo Clinic, Rochester, MN, United States

**Keywords:** head and neck cancer, salivary gland cancer, salivary duct carcinoma, adenocarcinoma, androgen receptor, androgen deprivation therapy, metastatic cancer

## Abstract

**Objectives:** There is a lack of effective therapy for recurrent or metastatic salivary gland carcinoma. Androgen deprivation therapy has demonstrated efficacy in cases of salivary duct carcinoma (SDC) and high-grade adenocarcinoma not otherwise specified (NOS) that express androgen receptor.

**Materials and Methods:** We conducted a single institution retrospective cohort study examining patients treated for recurrent/metastatic SDC or high-grade adenocarcinoma NOS of the salivary gland. Survival analyses were performed to assess for efficacy of first-line androgen deprivation therapy (ADT) vs. first-line conventional cytotoxic chemotherapy. Efficacy of salvage ADT was also assessed.

**Results:** Fifty-eight patients were reviewed. Thirty-five patients had recurrent/metastatic disease of which 28 had SDC (80%) and 7 had high-grade adenocarcinoma NOS (20%). Median overall survival for first-line ADT was 25 months compared to 25 months for first-line chemotherapy [RR 0.54 (0.23–1.28, *p* = 0.16)]. Patients treated with first-line ADT had a response rate of 45% (9/20) and patients treated with first-line chemotherapy had a response rate of 14% (2/14). Six patients received salvage ADT with 1 patient demonstrating complete response and 3 with stable disease as best response (clinical benefit rate 67%).

**Conclusion:** Overall survival for first line ADT and first line cytotoxic chemotherapy was comparable but response rates to first-line ADT were higher than those with first-line chemotherapy. Salvage ADT is active in recurrent/metastatic salivary gland carcinoma.

## Introduction

Recurrent and metastatic (R/M) salivary gland carcinomas present a unique clinical challenge given their heterogeneity of clinical behavior and the lack of effective systemic therapies (1). Certain subtypes of salivary gland cancer, including salivary duct carcinoma (SDC) and high-grade adenocarcinoma not otherwise specified (NOS), are known for an increased risk of distant metastases and shorter survival compared to other indolent subtypes, such as adenoid cystic carcinomas or acinic cell carcinomas. SDC is a rare subtype representing 1–3% of salivary gland carcinomas with 80% occurring in the parotid gland and 15% in the submandibular gland (2, 3). They tend to behave aggressively with 60% of patients presenting with locally advanced disease and 10% presenting with distant metastases (2). High-grade adenocarcinoma NOS is another uncommon aggressive subtype of salivary gland malignancy that is also associated with higher incidence of metastatic disease (3). The Mayo Clinic Rochester experience previously described 26 cases of SDC treated at Mayo Clinic from 1960 through 1989, one of the largest cohorts at that time. The median survival was only 50% at 3 years, and <20% at 5 years. More than 40% of the patients developed local recurrence and >60% of cases developed distant metastases (4).

Historically, platinum-based regimens have been used as first-line therapy for R/M salivary gland cancer based on clinical trial data, but response rates vary widely by histologic subtype and chemotherapy largely remains ineffective in most patients (5–10). In recent years, evidence has been emerging to support the use of androgen deprivation therapy (ADT) for salivary cancers that express the androgen receptor (AR) (11–15). Both SDC and adenocarcinoma can express the AR at varying rates, 70–100% of SDC and 21% of adenocarcinoma (2, 16). A gonadotropin-releasing hormone (GnRH) agonist or antagonist can be used to cease gonadal androgen production through reduced luteinizing and follicle-stimulating hormone levels. Due to an initial rise in androgen production with GnRH agonist use, combined androgen blockade (CAB) with the addition of an AR antagonist is often used.

Although ADT has been shown to be effective for AR-positive salivary gland cancer, it is still unknown how the activity compares to chemotherapy and whether it confers a survival benefit over chemotherapy. In this study, we have updated the Mayo Clinic Rochester experience with SDC and high-grade adenocarcinoma NOS and examine the efficacy of up front ADT on patient outcomes compared to conventional cytotoxic chemotherapy in the R/M setting. In addition, we report on the responses of salvage ADT in our patient population after progression on first-line ADT.

## Materials and Methods

We retrospectively reviewed the medical records for patients with biopsy proven SDC and high-grade adenocarcinoma NOS of the salivary glands at Mayo Clinic Rochester. A waiver for informed consent was obtained with IRB approval. Eligibility was restricted to patients managed at Mayo Clinic Rochester from 1990 to 2018. Patients with locally advanced disease, recurrent disease, and *de novo* metastatic disease were included. Demographic and clinical data were collected including patient age, sex, primary tumor site, tumor stage at diagnosis (based on American Joint Committee on Cancer TNM Staging for the Major Salivary Glands, 7th edition, 2010), treatment of the primary tumor, patterns of recurrence, site of recurrence, treatment of R/M disease, number of recurrences, and number of lines of systemic therapy. Surgical pathology archival material was histopathologically reviewed using hematoxylin and eosin-stained slides. Immunohistochemistry for human androgen receptor was performed on formalin-fixed, paraffin-embedded tissue sections with the AR27 clone (Leica Biosystems, Newcastle, United Kingdom) using the automated Ventana BenchMark XT (Roche Diagnostics). Cases were categorized as *androgen receptor-positive* if >50% of tumor cell nuclei were strongly positive for androgen receptor by immunohistochemistry. Pathological data including histology, presence of perineural invasion, presence of extraparenchymal extension, and androgen receptor status were recorded when available. Data was summarized with frequencies and percentages for categorical variables or means and standard deviations (or medians and ranges) for continuous variables. Baseline data for patients with R/M disease was compared to those who did not have R/M disease. Within the R/M cohort, the first-line ADT cohort was then compared to the first-line chemotherapy cohort. These comparisons were performed using the chi-square test or Fisher's exact test for categorical variables and using 2-sample *t*-test for continuous variables.

In order to calculate survival, recurrence, and response endpoints, we recorded the date of diagnosis (date of initial biopsy), date of first treatment, date of recurrence (date of biopsy if available; alternatively date of imaging, or clinic visit documenting recurrence), and date of death or last follow-up. First-line ADT was compared to first-line conventional cytotoxic chemotherapy in terms of overall survival (OS). OS was also secondarily analyzed for salvage ADT. Recurrence-free survival was assessed when able. Survival analysis was performed using the Kaplan-Meier method. OS was measured from date of first treatment for patients with *de novo* metastasis or date of recurrence for patients with recurrent disease following definitive therapy and the endpoint used was death from any cause. Patients alive at last known follow-up were censored. Relative risk was determined using the Cox proportional hazards model. All analyses were performed using JMP® Pro version 14.1.0 (SAS Institute Inc.). A *p*-value <0.05 was considered statistically significant. Response rates were determined by using best response to therapy based on provider assessment at that time.

## Results

Fifty-eight patients were included in the overall cohort. Forty-two patients (72%) had SDC and 15 patients (26%) had high-grade adenocarcinoma NOS. One patient (2%) had carcinoma ex pleomorphic adenoma with no clear recording of SDC or adenocarcinoma histology. Thirty-five patients (60%) developed R/M disease of which 28 had SDC (80%) and 7 had high-grade adenocarcinoma NOS (20%). Compared to patients who did not recur or develop metastatic disease, the patients with R/M disease had fewer females (*p* = 0.01), more SDCs (*p* = 0.05), more perineural invasion (*p* < 0.01), and more extraparenchymal extension (*p* = 0.01). They otherwise had similar ages, sites of disease, T & N stages, and androgen receptor status. Thirty-four patients (97%) were androgen receptor positive with one patient having AR negative adenocarcinoma.

Of the patients who developed R/M disease, 20 patients (57.1%) received first-line ADT of which 17 patients (85%) had SDC and 3 patients (15%) had high-grade adenocarcinoma NOS. Of the twenty patients who received first-line ADT, 7 patients had distant metastases at initial diagnosis and 13 developed R/M disease after initial curative-intent therapy. Fourteen patients with recurrent/metastatic disease (40%) received first-line cytotoxic chemotherapy of which 10 patients (71%) had SDC and 4 (28%) had high-grade adenocarcinoma NOS. Five patients had distant metastases at initial diagnosis and 9 patients developed R/M disease after completion of curative-intent therapy. One patient in the R/M cohort received first-line trastuzumab after recurrence.

Demographic, clinical and histopathologic data are summarized in Table 1. Age was statistically similar in both groups (*p* = 0.21); median ages were 63 and 66 for the first-line ADT and first-line chemotherapy groups, respectively. Both the first-line ADT group and first-line cytotoxic chemotherapy group were predominantly male (*n* = 20, 100% in first-line ADT group; *n* = 12, 86% in the first line chemotherapy group; *p* = 0.05). Tumors mostly occurred in the parotid gland in both groups (90 and 93%; first-line ADT and first-line chemotherapy, respectively). Tumor stage distribution was statistically similar between groups (T stage *p* = 0.56, N stage *p* = 0.66, M stage *p* = 0.15). There was no significant difference in histology between the two groups (*p* = 0.34). There was a significant difference between groups for perineural invasion (*p* = 0.03) with more in the ADT group having perineural invasion present on pathology (*n* = 13, 65%) compared to the chemotherapy group (*n* = 7, 50%). Extraparenchymal extension was similar between groups (*p* = 0.24). A median of 2 lines of systemic treatment were given in the metastatic setting for both groups (*p* = 0.15).

**Table 1:** Salivary duct carcinoma patient characteristics.

	**Metastatic/recurrent cohort**	**First-line androgen deprivation therapy**	**First-line chemotherapy**	***P*-value**
N	35	20	14	
Age				0.21
Mean (SD)	64.9 (13.2)	67.25 (10.1)	63.8 (14.6)	
Median	65	63	66	
Q1, Q3	59, 75	59, 77	60.25, 73	
Range	32–87	50–87	34–84	
Gender				0.05
Female	2 (6%)	0	2 (14%)	
Male	33 (94%)	20 (100%)	12 (86%)	
Site				0.97
Parotid	32 (91%)	18 (90%)	13 (93%)	
Submandibular	2 (6%)	1 (5%)	1 (7%)	
Could not be specified	1 (3%)	1 (5%)	0	
T stage				0.56
T1/T2	3 (9%)	3 (15%)	0	
T3/T4	15 (71%)	13 (65%)	14 (100%)	
TX	6 (17%)	4 (20%)	0	
Could not be determined	1 (3%)	0	0	
N stage				0.66
N0/N1	5 (15%)	5 (15%)	0	
N2/N3	20 (58%)	10 (50%)	9 (64%)	
NX	10 (29%)	5 (25%)	5 (36%)	
M stage				0.15
M0	11 (31%)	8 (40%)	2 (14%)	
M1	12 (34%)	7 (35%)	5 (36%)	
MX	12 (34%)	5 (25%)	7 (50%)	
Histology				0.34
Salivary duct carcinoma	28 (80%)	17 (85%)	10 (71%)	
High-grade adenocarcinoma NOS	7 (20%)	3 (15%)	4 (28%)	
Perineural invasion				0.03
Yes	20 (57%)	13 (65%)	7 (50%)	
No	7 (20%)	3 (15%)	4 (29%)	
Missing/Not applicable	8 (23%)	4 (20%)	3 (21%)	
Extraparenchymal extension				0.24
Yes	23 (66%)	12 (60%)	10 (71%)	
No	5 (14%)	4 (20%)	1 (7%)	
Missing/Not applicable	7 (20%)	4 (20%)	3 (21%)	
Androgen staining				0.23
Positive	34 (97%)	20 (100%)	13 (93%)	
Negative	1 (3%)	0	1 (7%)	
Lines of systemic treatment in metastatic setting				0.15
Mean (SD)	2.3 (1.8)	2.2 (1.6)	2.2 (1.8)	
Median	2	2	2	
Range	1–7	1–7	1–7	

**Figure 1:**
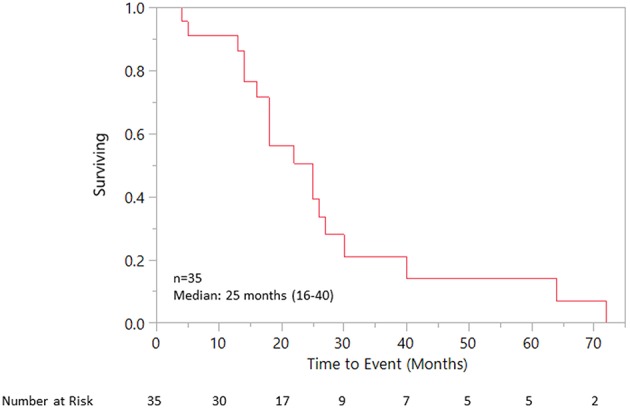
Overall survival for recurrent/metastatic cohort.

**Figure 2:**
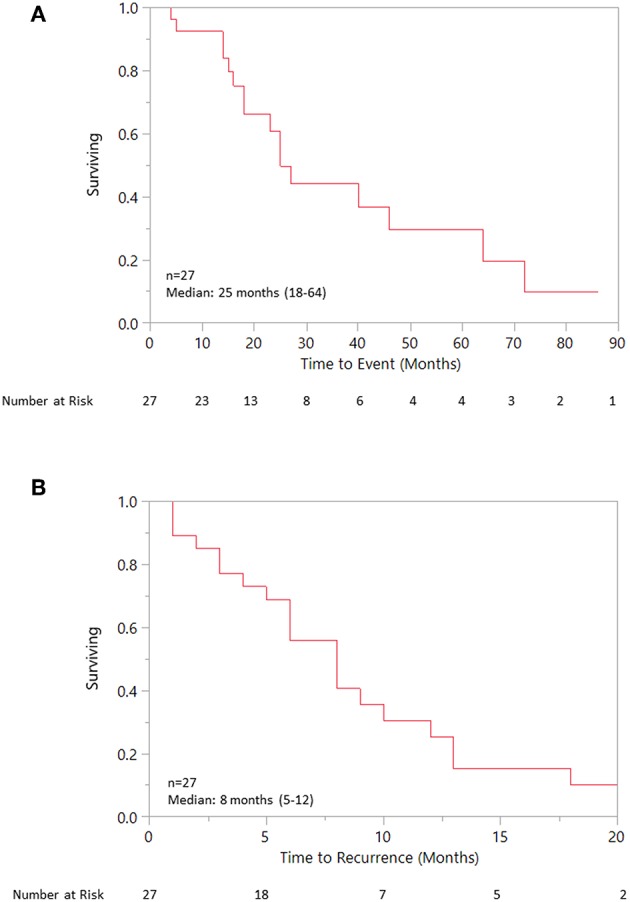
**(A)** Overall survival for patient receiving any ADT. **(B)** Progression free survival for patient receiving any ADT.

**Figure 3:**
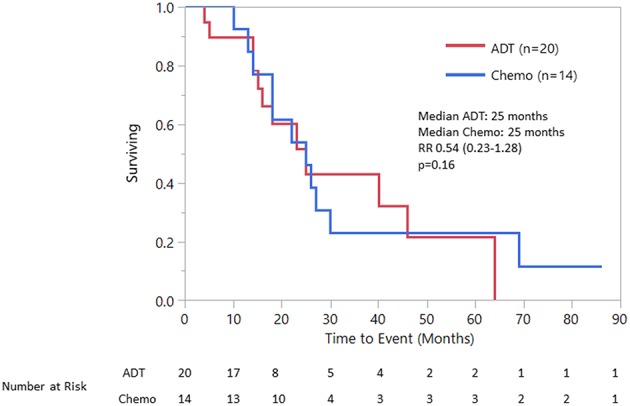
Overall survival first line ADT vs. First line chemotherapy.

**Figure 4:**
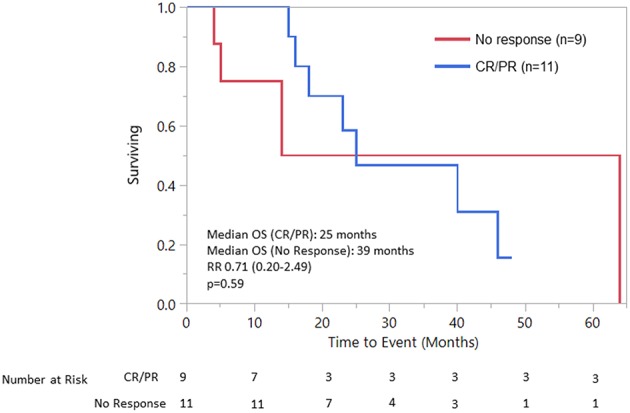
OS response to 1st line ADT vs. No response to 1st line ADT.

Median OS was 25 (18–40) months specifically for the recurrent/metastatic cohort (Figure 1). Twenty-seven patients (46%) received ADT sometime during their course of palliative treatment and had a median OS of 25 (18–64) months and median time to progression of 8 (5–12) months (Figures 2A,B). When comparing first-line ADT to first-line cytotoxic chemotherapy, median OS was 25 months for both groups with RR 0.54 (CI 0.23–1.28, *p* = 0.16; Figure 3). Progression-free survival could not be compared due to missing data for many patients in the first-line chemotherapy cohort. Of patients receiving first-line ADT for R/M disease, 14 (70%) received combined androgen blockade (leuprolide and bicalutamide), 1 (5%) received leuprolide monotherapy, 2 (10%) received bicalutamide monotherapy, and 3 (15%) received enzalutamide monotherapy. In total, 11 of 20 patients (55%) with R/M AR-positive SDC or adenocarcinoma had at least a partial response to ADT with an 8 (1–19) month median duration of response. The response rate by histologic subtype was 53% for SDC and 67% for adenocarcinoma. For the 11 patients that had an objective response to first-line ADT, the median overall survival was comparable to the 9 patients that did not have objective response with RR 0.71 (CI 0.20–2.49, *p* = 0.59; Figure 4). Median OS for 5 patients that specifically progressed on first-line ADT was 14 months. Best response after treatment could not be determined in 3 cases. There was one case of stable disease with first-line enzalutamide therapy that persisted for 6 months. In the first-line chemotherapy cohort, 13 out of 14 patients (93%) received platinum-based chemotherapy with regimens consisting of carboplatin/paclitaxel, cisplatin/5-flurouracil, cisplatin/etoposide, carboplatin/vinorelbine, or mitomycin-C/cisplatin/vinblastine. The only non-platinum based chemotherapy used was capecitabine monotherapy. There were 2 complete responses (1 SDC and 1 adenocarcinoma) and 0 partial responses for a response rate of 14% (2/14). There were 3 cases of stable disease persisting for 4, 6, and 11 months.

In patients receiving >1 line of systemic treatment, 6 patients were treated with salvage ADT after progression on first-line ADT and had a median overall survival of 25 months. Of the six patients who received salvage ADT following first-line ADT, the responses were as follows: 1 complete response, 0 partial responses and 3 stable disease for a clinical benefit rate of 67% (4 of 6). The patient who had a complete response to salvage ADT first received enzalutamide with stable disease for 6 months, then was immediately treated with salvage leuprolide resulting in a persistent complete response. Three patients with stable disease as best response were on salvage antiandrogen treatment. The first case involved a patient with SDC with bone metastases at initial presentation who achieved a partial response to first-line bicalutamide at 1 month. Second-line treatment with combined leuprolide and bicalutamide was then immediately used resulting in stable disease for 13 months. The second case also involved a patient with bone metastases at initial diagnosis from a high-grade adenocarcinoma NOS that had an initial complete response to first-line combined leuprolide and bicalutamide for 16 months. Following progression, second-line leuprolide and abiraterone was used 2 months later resulting in stable disease for 23 months. The third case involved recurrent SDC initially treated with combined leuprolide and bicalutamide followed by progressive disease after 7 months of therapy. Second-line enzalutamide was then used 1 month later resulting in stable disease for 7 months. There were 2 cases of progressive disease despite salvage ADT. One case initially received leuprolide and bicalutamide first-line with complete response for 12 months and then was switched to salvage abiraterone 1 month later with progression after 2 months. The second case initially received enzalutamide monotherapy with progression after 1 month and then was immediately treated with salvage leuprolide and bicalutamide with progression after 1 month.

Of the 14 patients who received first-line chemotherapy, 6 patients (42%) went on to receive ADT following progression. No patients received chemotherapy alone as second-line following first-line ADT. Of the 6 patients that received ADT following initial chemotherapy, 1 had a partial response and one had stable disease for a response rate of 16.7% and a clinical benefit rate of 33%. In the responsive case, the patient initially received six cycles of carboplatin and paclitaxel chemotherapy for SDC with *de novo* bone metastases resulting in stable disease for 4 months. Combined androgen blockade with leuprolide and bicalutamide was then used resulting in a partial response for 8 months. In the case of stable disease, the patient had a prolonged duration of stable disease (40 months) with second-line leuprolide and bicalutamide following an initial complete response to platinum-based chemotherapy for metastatic SDC. See Table 2 for a summary of first-line and salvage ADT regimens with associated responses.

**Table 2:** Androgen Deprivation Therapy (ADT) regimens and response rates.

**Treatment regimen**	**Response rate**
First-line ADT (*n* = 20)	55%
Leuprolide + Bicalutamide (*n* = 14)	5 CR, 4 PR, 4 PD, 1 Unknown
Leuprolide (*n* = 1)	1 PD
Bicalutamide (*n* = 2)	2 PR
Enzalutamide (*n* = 3)	1 SD, 2 PD
Salvage ADT after 1st line ADT (*n* = 6)	16.7%
Leuprolide + Bicalutamide (*n* = 2)	1 SD, 1 PD
Leuprolide (*n* = 1)	1 CR
Enzalutamide (*n* = 1)	1 SD
Leuprolide + Abiraterone (*n* = 1)	1 SD
Abiraterone (*n* = 1)	1 PD
Salvage ADT after 1st line Chemo (*n* = 6)	16.7%
Leuprolide + Bicalutamide (*n* = 2)	1 PR, 1 SD

*CR, complete response; PR, partial response; SD, stable disease; PD, progressive disease*.

## Discussion

This retrospective study compared ADT to conventional chemotherapy for recurrent or metastatic salivary gland cancer, specifically in SDC and adenocarcinoma subtypes. This study provides an update to the previously published study of the Mayo Clinic experience with SDC, and confirms that ~60% of patients with SDC will ultimately develop R/M disease. Despite the high number of recurrences, patients are living on average 2 years with R/M disease, illustrating the variable clinical behaviors and biology seen with R/M SDC (and adenocarcinoma). Our results demonstrated comparable survival for patients who received first-line ADT compared with first-line chemotherapy, although the results are confounded by small numbers, heterogeneous clinical courses, and the fact that 42% of patients received ADT after failure of chemotherapy. Notably, the response rate for first-line ADT was markedly higher than that of first-line chemotherapy (45 vs. 14%). The selection of first-line therapy (ADT vs. chemotherapy) was mainly based on availability of AR testing at the time of the patient encounter as opposed to performance status and, as a result, those who received chemotherapy first-line were treated in earlier years than those receiving first-line ADT. Patients who specifically progressed after first-line ADT had a lower median OS compared to those who had objective response (14 vs. 25 months) indicating that progression could be a predictor of poorer outcomes as well as hormone resistant disease and deserves further study.

Since the discovery of AR expression in SDC and high-grade adenocarcinoma NOS, several studies have explored the efficacy of ADT. A case series of patients with salivary gland tumors reported an overall response rate of 64.7%, including three complete clinical responses, with combined androgen blockage and inhibition of androgen receptor signaling for salivary gland tumors (11). A small case series specifically examining patients with SDC described a median progression-free survival of 12 months with therapy directed toward the androgen receptor (12). Patient response to multiple sequential lines of androgen blockade despite progression has also been described (13). The largest androgen receptor specific study to date involved a Netherlands cohort of 35 patients with SDC and reports a median progression free survival of 4 months and median overall survival of 17 months (14). The first prospective study, a phase II trial of 34 patients with SDC treated with leuprolide and bicalutamide, cites a progression-free survival of 8.8 months and median overall survival of 30.5 months (15). Our study demonstrates comparable overall survival to these previous reports in the literature.

Chemotherapy remains a challenging and unreliable treatment for R/M salivary cancer. Our study shows modest responses to chemotherapy that are consistent with the published literature. The only randomized study of chemotherapy for salivary cancer was an Italian phase II study that demonstrated superiority of cisplatin and vinorelbine over vinorelbine alone with 19% complete response and 25% partial response for cisplatin and vinorelbine compared to 0% complete response and 20% partial response for vinorelbine alone. However, there was no difference in survival and this study included all histologies with SDC serving as a small part of the cohort (8). A follow-up prospective cohort study of combination vinorelbine and cisplatin showed better response rates when used as first-line therapy, specifically in adenocarcinoma (9). Our study is valuable as it provides data on response rates to chemotherapy for AR-positive SDC and adenocarcinoma specifically.

Despite prior studies examining the efficacy of various chemotherapeutic regimens and ADT, no published study to date has compared the two. There is an ongoing EORTC phase II randomized study of ADT vs. chemotherapy in patients with AR-positive salivary cancer which will further inform this question (NCT01969578). Our study suggests a benefit for salvage ADT in those patients who respond to first-line ADT with a clinical benefit rate of 67%. The results of the completed Alliance study of enzalutamide for AR-positive salivary cancer have recently reported 1 partial response, 7 stable disease, and 3 progressive disease for 11 patients who were previously treated with ADT (NCT02749903).

Limitations of our study included the retrospective nature of the study itself introducing potential bias and missing data points. The standard use of first-line ADT for AR positive patients at our institution required the addition of many patients primarily treated in the 1990s to represent the first-line chemotherapy cohort. This led to missing data in our latest electronic medical records in regards to specific events following treatment aside from date of death or last follow-up which prohibited any meaningful PFS calculations. Given the rarity of the disease, our analysis is limited by small numbers for comparison. Further investigation of ADT as first-line and salvage treatment is warranted.

## Data Availability

All datasets generated for this study are included in the manuscript and/or the supplementary files.

## Ethics Statement

This study was approved by the Mayo Clinic Institutional Review Board (IRB).

## Author Contributions

PV, KP, JG, DS-W, and AC all contributed to the study design, data collection, statistical analysis, and manuscript development. All authors have reviewed the manuscript and are in agreement with submission.

### Conflict of Interest Statement

The authors declare that the research was conducted in the absence of any commercial or financial relationships that could be construed as a potential conflict of interest.
